# A Patch/Dipole Hybrid-Mode Antenna for Sub-6GHz Communication

**DOI:** 10.3390/s19061358

**Published:** 2019-03-18

**Authors:** Bei Huang, Weifeng Lin, Jialu Huang, Jun Zhang, Gary Zhang, Fugen Wu

**Affiliations:** 1 School of Materials and Energy, Guangdong University of Technology, Guangzhou 510006, China; 1111702006@mail2.gdut.edu.cn (B.H.); wufg@gdut.edu.cn (F.W.); 2 School of Information Engineering, Guangdong University of Technology, Guangzhou 510006, China; linweifeng@mail2.gdut.edu.cn (W.L.); pber@outlook.com (J.H.); garyzhang@gdut.edu.cn (G.Z.)

**Keywords:** hybrid mode, slotted patch, low profile, Sub-6GHz communication

## Abstract

A low-profile antenna with a high gain and broad bandwidth is proposed for Sub-6GHz communication in this paper. A narrow-band patch mode and a narrow-band dipole mode are shared in one radiator and simultaneously excited to broaden the bandwidth. A compact prototype with a projection size of 0.90 λ_0_ × 0.78 λ_0_ and a profile of 0.13 λ_0_ (λ_0_ is the wavelength in the free space at the center of the operating frequency) is fabricated and measured. The measurement demonstrates an impedance bandwidth of 67.50%, covering the frequency range from 2.75 GHz to 5.45 GHz and an average gain of 8.4 dBi in the operating band of 3.0–5.0 GHz.

## 1. Introduction

Microstrip antennas are popular structures for a shared aperture due to their low mass and ability to be easily integrated with the feeding network [1], which are suitable for micro base station applications for persuasive fifth generation communication. Dipoles and microstrip patches were interlaced in [2] to realize a bandwidth of 8.9% in the S-band and 17.0% in the X-band, with a frequency ratio of 1:3. A dual-band dual-polarized antenna was introduced in [3], where a parasitic patch was stacked over a driven patch. In this way, each polarization could share one feeding network, which contributed to the reduction of the feeding networks and input ports. Therefore, the antenna had two operating bands, respectively covering the C-band from 5.05 to 5.30 GHz and the X-band from 9.60 to 10.30 GHz.

Both the patch and dipole have a small bandwidth within a limited profile. A common method is to shape the patch or slot for broadening the bandwidth. Two conventional quarter-wave patch antennas with an L-slit and U-slot were implemented and compared in [4], which achieved impedance bandwidths of 45.0% and 53.0%, with profiles of less than 0.13 λ_0_ and 0.11 λ_0_, respectively. Here, λ_0_ is the wavelength in the free space at the center of the operating frequency. However, an increase in bandwidth using slotting techniques is always at a sacrifice of antenna gain. Therefore, how to keep a large bandwidth and a high gain at the same time is an attractive issue for a compact element.

A magneto-electric (ME) dipole antenna was proposed in [5], where an obtuse-triangular structure was used as a magnetic dipole. The ME dipole could reduce the profile to 0.097 λ_0_, maintaining a stable gain of 9.2 ± 1.1 dBi and achieving an impedance bandwidth of 28.2%. Hybrid mode is another promising candidate through sequentially exciting the adjacent modes in the interested band [6]. Two adjacent modes could be simultaneously excited by a crossed bow-tie dipole and an octagonal-ring antenna [7]. It retains the advantages of a low profile, broad bandwidth, and stable radiation pattern.

Similarly, the hybrid mode with sidewalls for the lower ultra-wide band was proposed in [8], in which the patch mode and slot mode were sequentially excited in a shared aperture. This antenna could cover a frequency band from 3.00 to 4.99 GHz and maintain a minimum gain of 9.0 dBi with a projection area of 1.09 λ_0_ × 1.04 λ_0_ and a profile of 0.13 λ_0_.

This paper is an extension of the conference paper [9], where mode analysis is detailed to demonstrate the mode hybridization. In conjunction with a tapered feeding line, a broadband balun is designed to convert the single feeding to differential feeding. In this way, the complexity of the feeding method is reduced when compared with the hybrid mode antennas [7,8]. The low-cost substrate used can also help to reduce the cost of this antenna and therefore support the dense deployment for micro base station applications. The frequency bands of n77 (3300–4200 MHz), n78 (3300–3800 MHz), and n79 (4400–5000 MHz), specified by the Third Generation Partnership Project (3GPP) for 5G communication, are covered by the proposed antenna for Sub-6GHz communication [10].

## 2. Antenna Design and Analysis

**Figure 1:**
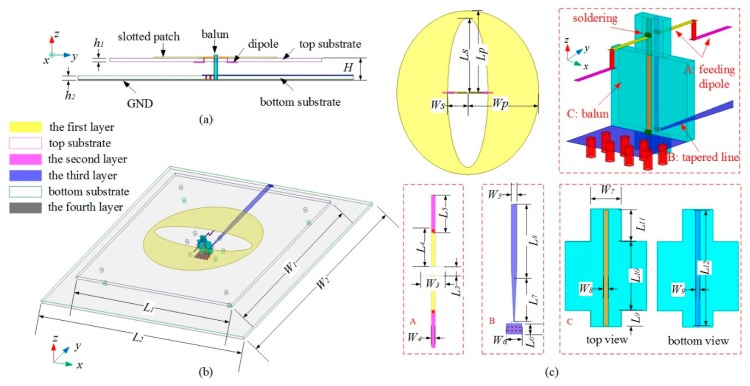
Geometry of the proposed antenna (**a**) side view, (**b**) perspective view, and (**c**) zoom in (part A: the feeding dipole, part B: the tapered line, and part C: both sides of the balun).

Figure 1 presents the geometry of the proposed antenna, which consists of an elliptical slotted patch, a feeding dipole connected with a broadband balun, and top and bottom substrates. The elliptical slotted patch is printed on the top side of the top substrate, which has been demonstrated to operate in an ultra-wideband for unidirectional radiation due to its tapered structure [11]. To ease the assembly and soldering, the feeding dipole is split into two parts on the top and bottom sides of the top substrate and these two parts are connected by the plating through holes. Two vertical parallel feeding lines, i.e., the balun structure, are designed to differentially excite the feeding dipole. The impedance transformation is implemented by a tapered line and printed on the top side of the bottom substrate. The ground plane is printed on the bottom side of the bottom substrate. All substrates used are FR4 materials.

Four parameters are used to describe the geometry of the slotted patch, namely, the halves of the long axis and short axis of the elliptical slot (*L_S_*, *W_S_*), and those of the elliptical patch (*L_P_*, *W_P_*). The elliptical patch is tuned to operate at its fundamental mode in the lower frequency band at its initial design. With an elliptical slot etched on its center, each arm of the feeding dipole has a length of about a quarter of the wavelength [8]. By properly selecting the value, the hybrid mode can be sequentially excited in the band of interest. The detailed dimensions of the proposed antenna are listed in Table 1. The sizes of the top and bottom substrates are 100 mm × 100 mm and 130 mm × 130 mm, respectively. The profile of the antenna is 10 mm.

**Figure 2:**
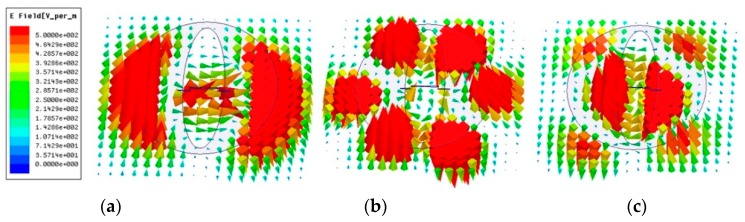
The simulated electric field distribution of the proposed antenna without a feeding network and balun (remove the feeding network and balun, fed in the center of the feeding dipole by a lumped port) at (**a**) 3.0 GHz, (**b**) 4.0 GHz, and (**c**) 5.0 GHz.

**Figure 3:**
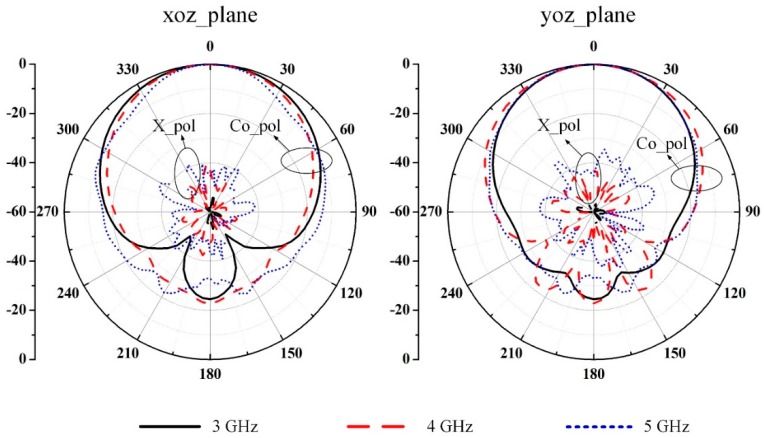
Simulated radiation patterns of the proposed antenna without a feeding network and balun at (**a**) *xoz* plane and (**b**) *yoz* plane.

For better understanding the operation of the proposed antenna, the electric field distribution underneath the radiator is extracted and displayed in Figure 2. The feeding network and balun are removed for more distinct modes and the antenna is excited at the center of the feeding dipole. The port is excited with an identical unit input power throughout all frequencies. The same scale is shared on the left. It is found that the dipole plays an important role in the higher frequency region. The patch mode dominates the radiation over the operating frequency range. Additionally, the slotting technique in the center of the patch can help to suppress the cross-polarization level [12]. Figure 3 shows the simulated radiation patterns without the feeding network and balun. In the operating frequency band of 3.0 to 5.0 GHz, both *xoz* and *yoz* planes can maintain stable radiation patterns in terms of half-power beam-widths (HPBWs). More to the point, the HPBWs are 68.40°, 67.98°, and 58.36° in the *xoz* plane and 56.02°, 57.46°, and 41.62° in the *yoz* plane at 3.0, 4.0, and 5.0 GHz, respectively.

## 3. Results and Discussions

**Figure 4:**
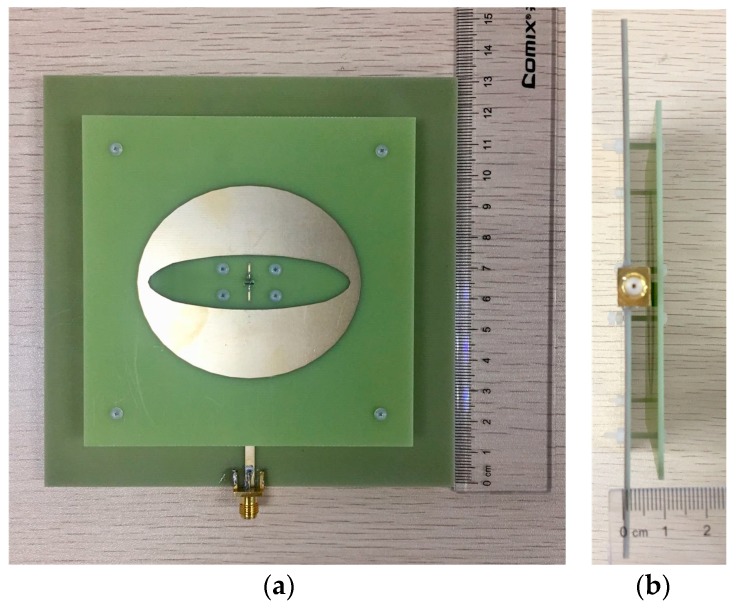
Photograph of the antenna prototype: (**a**) top view and (**b**) side view.

**Figure 5:**
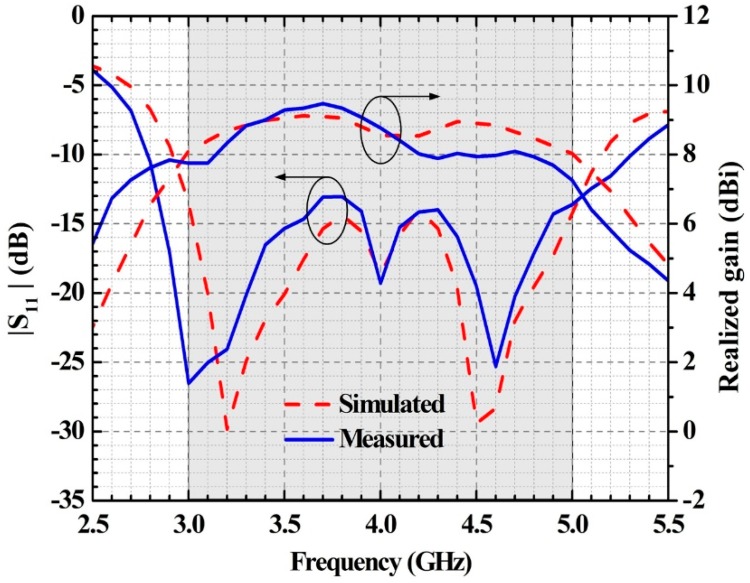
The simulated and measured reflection coefficients and realized gains of the proposed antenna.

Figure 4 shows the photograph of the prototype antenna. Nylon screws are used to fix the top and bottom substrates. The reflection coefficients of the prototype are measured by the KEYSIGHT E5071C Vector Network Analyzer (Keysight Technology, New York, USA). The radiation patterns and gains are measured in an anechoic chamber. Figure 5 illustrates the simulated and measured reflection coefficients and broadside gains versus frequency. The simulated 10-dB impedance bandwidth is 55.75%, covering 2.92 GHz to 5.15 GHz. The measured 10-dB impedance bandwidth is 67.50% in the range of 2.75 GHz to 5.45 GHz. Therefore, the impedance bandwidth of the proposed antenna can cover the frequency bands of n77 (3300–4200 MHz), n78 (3300–3800 MHz), and n79 (4400–5000 MHz).

The simulated radiation efficiency of the proposed antenna varies from 70% to 80% within the operating band, where the minimum value is reached at 5.0 GHz. The simulated gain varies between 8.0 and 9.1 dBi with 1.1-dB variation, while the measured one varies between 7.3 and 9.5 dBi, with 2.2-dB variation. The discrepancies between the simulated and measured results can be attributed to fabrication and assembly errors.

**Figure 6:**
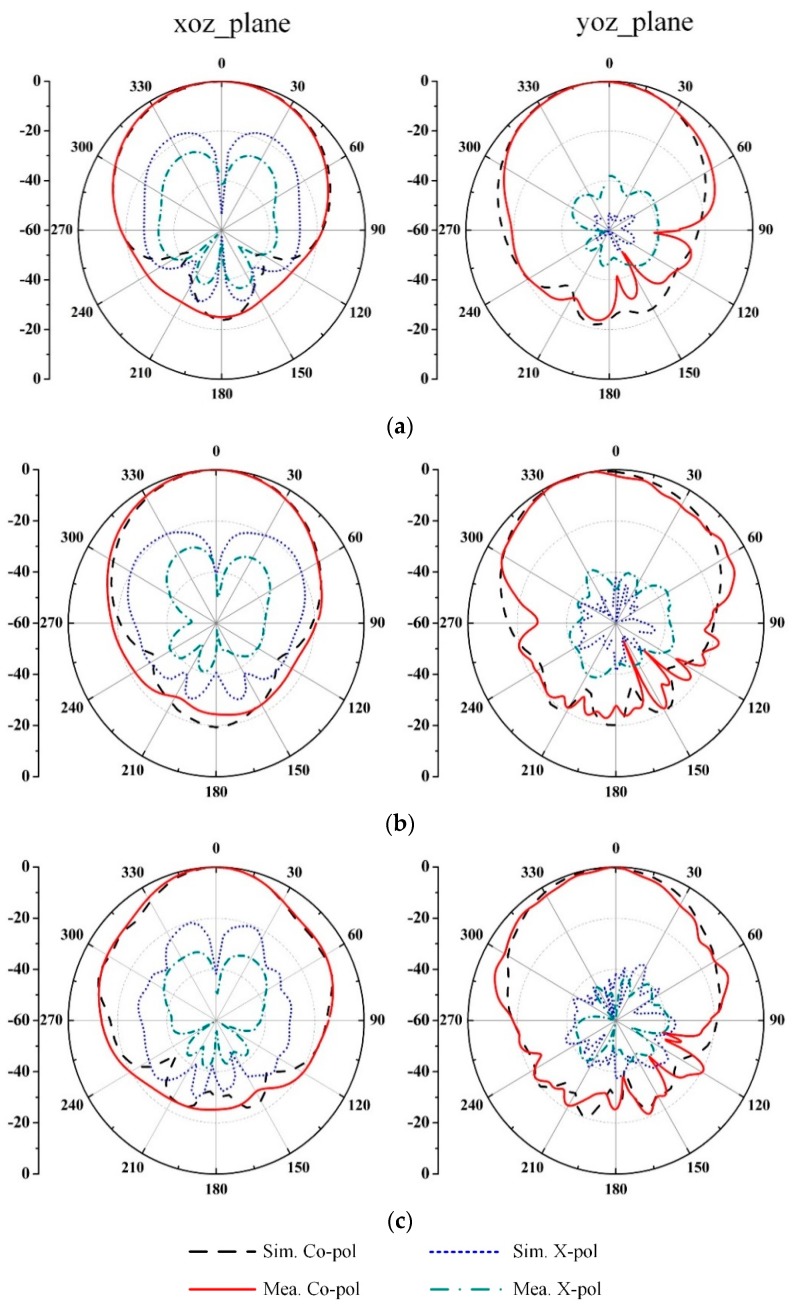
Simulated and measured radiation patterns of the proposed antenna at (**a**) 3.0 GHz, (**b**) 4.0 GHz, and (**c**) 5.0 GHz.

The radiation patterns of the prototype antenna at three typical frequencies are displayed in Figure 6. The measured radiation patterns in both *xoz* and *yoz* planes agree with the simulated ones. The HPBWs are 70.4°, 55.7°, and 43.2° in the *xoz* plane and 46.3°, 43.3°, and 40.5° in the *yoz* plane at 3.0 GHz, 4.0 GHz, and 5.0 GHz, respectively. However, the spurious radiation from the asymmetric balun [13] causes a slight shift of the main beam from the broadside direction in the *yoz* plane. Meanwhile, the polarization purity of the proposed antenna is deteriorated at the *xoz* plane. The simulated cross polarization in the worst case is less than −18.5 dB over the operating band, while the measured one is around −28.5 dB in the operating frequency band. The difference in the cross polarization may be caused by assembly and measurement errors.

A comparison of the proposed antenna and gain-bandwidth enhanced antennas in the literature is summarized in Table 2. Ref. [5] has a lower profile and higher antenna gain, but a narrower bandwidth. The reduction in the profile but increase in the size is obvious when compared with [14]. Though [15] and [16] have a much lower profile, the proposed antenna has a larger bandwidth or compacter size. Moreover, the radiation pattern in [16] has side lobes, which may cause the reduction in the directivity.

## 4. Conclusions

An elliptical patch/dipole antenna operating in a hybrid mode has been designed and investigated to simultaneously enhance the gain and bandwidth in a low profile. The bandwidth is about 67.50%, covering the frequency from 2.75 GHz to 5.45 GHz. An average gain of 8.40 dBi in the operating band of 3.0–5.0 GHz is achieved with a projection size of 0.90 λ_0_ × 0.78 λ_0_ and a profile of 0.13 λ_0_. The proposed antenna has a simple structure and low fabrication cost. The antenna may find its applications in the micro base station for Sub-6GHz communications. The distortion of the radiation pattern resulting from the spurious radiation of the balun [17] will be addressed in the future.

## Figures and Tables

**Table 1:** Dimensions of the Proposed Antenna.

**Parameter**	*L*_1_	*L*_2_	*L*_3_	*L*_4_	*L*_5_	*L*_6_	*L*_7_	*L*_8_	*L*_9_	*L*_10_	*L*_11_	*L*_12_	*h*_1_	*h*_2_
**Value (mm)**	100.00	130.00	1.20	5.20	5.20	5.80	29.40	35.00	1.60	6.80	3.20	11.40	1.60	1.60
**Parameter**	*W*_1_	*W*_2_	*W*_3_	*W*_4_	*W*_5_	*W*_6_	*W*_7_	*W*_8_	*W*_9_	*L_S_*	*W_S_*	*L_P_*	*W_P_*	*H*
**Value (mm)**	100.00	130.00	3.20	0.50	2.92	8.00	3.00	0.50	0.42	30.75	8.35	33.75	29.25	10.00

**Table 2:** Comparison with Gain-Bandwidth Enhanced Antennas.

Reference	Projection Size (λ_0_ × λ_0_)	Profile (λ_0_)	Bandwidth	Realized Gain (dBi)	Cross Polarization (dB)	Front-to-Back Ratio (dB)	HPBW (°) ^#^	
							*xoz* Plane	*yoz* Plane
[5]	0.93 × 0.51	0.097	28.2%	9.2 ± 1.1	−24.0	13.0	42.0	46.0
[14]	0.62 × 0.62	0.24	68.0%	8.1 ± 1.5	−23.0	14.0	56.0	53.0
[15]	0.82 × 0.69	0.06	28.4%	8.2 ± 0.9	−25.0	13.0	40.0	44.2
[16]	1.02 × 1.31	0.06	55.0%	7.0 ± 3.0	−25.2	17.0	24.2	34.3
This work	0.90 × 0.78	0.13	67.5%	8.4 ± 1.1 *	−28.5	26.5	55.7	43.3

^#^ The value of the half-power beam-width (HPBW) is extracted at the center operating frequency; * The radiation performance is extracted from 3.0 GHz to 5.0 GHz.
